# Iron deficiency anemia in children: a challenge for public health and for society

**DOI:** 10.1590/S1516-31802005000200011

**Published:** 2005-03-02

**Authors:** Geraldo Gaspar Paes Leme Coutinho, Eny Maria Goloni-Bertollo, Érika Cristina Pavarino Bertelli

**Keywords:** Public health, Iron deficiency anemia, Child, Strategies, Saúde pública, Anemia ferropriva, Criança, Estratégias

## Abstract

Iron deficiency anemia is the principal nutritional dearth in the world, and it especially affects children and pregnant women in developing countries. This paper presents a survey of the literature in this area, with the aim of providing a brief overview regarding the occurrence of iron deficiency anemia in Brazil. The article describes the etiology of the disease, the risk groups, the high prevalence of anemia in several areas of Brazil, and also the consequences of iron deficiency in children. The paper also shows some ways to control iron deficiency anemia and some intervention programs applied in Brazilian cities for curing and/or preventing this disease. The article concludes by emphasizing the need to establish strategies and treatments in our country that are based on a policy that brings together not only governmental administration but also all the community.

## RESUMO

A anemia ferropriva é a principal carência nutricional no mundo, com etiologia relacionada a múltiplos fatores, e afeta crianças e gestantes principalmente nos países em desenvolvimento. O presente artigo apresenta uma revisão da literatura na área visando oferecer um panorama sucinto da ocorrência de anemia ferropriva no Brasil. O texto mostra a etiologia dessa doença, quais são os grupos de risco, sua alta prevalência em algumas regiões do Brasil e as conseqüências da carência de ferro em crianças. Além disso, procurando apontar alguns caminhos para a erradicação dessa doença, o artigo aborda as formas de controle da anemia ferropriva, bem como alguns programas preventivos e/ou de tratamento que foram ou estão sendo implementados em municípios brasileiros. Os autores concluem enfatizando a necessidade de serem multiplicados, em nosso país, esses tipos de programas a partir de uma política que envolva não só os poderes públicos, mas também toda a comunidade.

## INTRODUCTION

Nutritional anemia, according to the World Health Organization (WHO), is a state in which the hemoglobin concentration in the blood is lower than levels considered normal for the age, gender, physiological state and altitude, as a consequence of shortage of essential nutrients, independent of the cause of this deficiency.^1,2^ Nutritional anemia includes a lack of nutrients such as iron, folic acid, vitamin B_12_ and copper (with erythropoietic function), vitamins C and E (related to hemorrhagic states) and vitamin A (related to cellular differentiation of red blood cells and mobilization of the iron of the reticuloendothelial system).^3^

The occurrence of anemia due to iron deficiency is denominated iron deficiency anemia.^4^ This deficiency is the most common nutritional disorder in infancy, and it affects communities not only in developing nations but also in highly industrialized countries.^5,6^ Iron deficiency anemia also affects women of childbearing age. These two groups are the ones most affected by shortages of minerals.^7,8^

### Etiology and risk groups

The main factors involved in the etiology of anemia in infants under 2 years of age are the iron reserves at birth, growth rate, diet and iron loss.^9^ Organic physiological loss of iron occurs in bile, urine and cellular desquamation of the skin and intestinal lining. In children, loss also occurs due to blood in the feces and by the use of whole milk in liquid form during the first year of life.^10,11^ Another possible cause of iron loss is the presence of intestinal parasites, although several studies have shown that the majority of parasitic diseases have secondary importance in the etiology of iron deficiency anemia in under 5-year-olds.^12–16^

The most significant weight gain and storage of iron by the fetus occurs during the last trimester of pregnancy. Premature births, intrauterine growth restriction and multiple pregnancies are factors that lead to iron deficiency anemia within the first six months of life, caused by low stocks of iron at birth.^6,10^ The average iron concentration per kilogram of body weight at birth is 70 mg/kg for full-term infants.^6,10^ The average daily iron needs are from 0.72 mg to 0.46 mg for children from five months to one year old and from one to three years old, respectively.^17^

In the diet, the quantity of bioavailable iron is important, and this is determined by stimulation and inhibitory factors that exist within a meal.^18^ Among the iron absorption stimulation factors in the diet are organic acids, in particular ascorbic acid, which is found in citric fruits.^18^ Among the iron absorption inhibitory factors are phytic acid, which is found in fibers, whole grains and beans,^19^ oxalic acid, which is found in spinach and beetroot,^20^ and tannin, which is found in tea, coffee and chocolate.^21^ Calcium, which is present in milk and dairy products,^22^ and other minerals that are close to iron in the periodic table, which compete with the same intestinal absorption,^23^ also inhibit the absorption of iron.

For full-term newborn babies, the iron deposits at birth provide the needs for this mineral until four to six months of age. Breastfeeding alone acts as a protective factor during the first months of life and, in spite of the low iron content of human milk (0.26 to 0.73 mg/l), the mineral in mother's milk has high bioavailability and absorption (around 50%).^24^ Because of the greater physiological requirements within the first two years of life, and specifically from 6 to 12 months because of the accelerated growth during this period, it is rare that the child will manage to ingest the recommended daily amount of iron. This is true even when good sources of bioavailable iron are introduced into the diet. Thus, preventive iron supplements are usually necessary for this age group.^17,25^

### Prevalence

According to the United Nations Children's Fund (Unicef), 90% of all types of anemia in the world are due to iron deficiency.^4^ In South and Central America, iron deficiency anemia has been a severe public health problem, affecting as many as 50% of pregnant women and children.^7^

In Brazil, studies performed in the State of São Paulo on almost 3,000 infants under 24 months of age, and in the State of Pernambuco on 777 children aged between 6 and 59 months, have demonstrated iron deficiency anemia rates of 59.1% and 40.9% respectively.^26,27^ In the city of São Paulo, a study performed on 1,015 children aged between 4 and 59 months reported prevalence of more than 49%, and of approximately 68% within the 6 to 23-month age range.^5^ Another Brazilian study performed in the city of Pontal, State of São Paulo, on 115 children aged from 12 to 72 months, confirmed prevalence of 68.7%.^28^ In the city of Porto Alegre in the State of Rio Grande do Sul, the prevalence of anemia among 12 to 23-month-olds was 65.6%.^29^ Coutinho found a prevalence of 75% among 6 to 24-month-old children in a district of the city of São José do Rio Preto, in the State of São Paulo.^30^

Studies performed in other regions of Brazil have revealed great variation in their results, finding prevalences of iron deficiency anemia ranging from 13.3% to 60.5% among preschool-age infants. These studies have also confirmed that the greatest rates were observed among infants under 24 months of age (41% to 77%).^31^ Recently, a study performed in the municipal of Goiânia, State of Goiás, on 110 breastfed babies aged from six to 12 months, who were born at full term and were not receiving iron sulfate supplements, demonstrated anemia prevalence of 60.9%.^32^

### Consequences of iron deficiency in children

Studies show that children with iron deficiency present worse performance in psychomotor tests than do non-anemic children.^33,34^ The greatest prevalence of iron deficiency among breastfed infants coincides with the final period of rapid brain development (six to 24 months), when the motor and cognitive skills take shape.^35^ Long-term prospective studies have also identified persistent cognitive deficiencies in 10-year-old children who had suffered from anemia during the first months of infancy.^36^

In South Africa, six to eight-year-olds who were observed to have low iron reserves, presented with retarded growth in comparison with those who had normal reserves.^37^ A boost in the growth of iron-deficient preschool children was seen after supplementation of this mineral.^38^ Also, 12 to 18-month-old children with iron deficiency presented the same rate of psychomotor development as did non-anemic children, after four months of treatment with iron supplements.^34^

Iron deficiency can also negatively affect cellular immunity, even before the child becomes anemic, and this can lead to an increase in illnesses such as diarrhea, respiratory disease and other infections. These effects can be reduced by iron supplementation or food fortification.^39,40^

### Forms of control

Despite the efforts by the World Health Organization (WHO), with their disease eradication programs, most of the world still faces difficulties in eliminating public health problems. In Brazil, the 8^th^ National Health Conference, which took place in 1987, accepted that health depends, among other things, on the state of the diet, housing, environment, salary, transport, leisure, liberty and access to healthcare services. This stance diverts attention from the disease as presented to the doctor, and leads to consideration of the human being as a whole and his living conditions. Thus, the emphasis in the debate on health is redirected from the purely biomedical and curative viewpoint to a broader, preventive view involving social and educational aspects.^41^

Additionally, the debate on health as a basic human right has been reopened. To this, a special emphasis on children's rights to health should be added, because of their immaturity and consequent incapacity for self-determination and defense, and emphasis on the responsibility of governments to guarantee and protect this right.^41^

Starting from this premise and considering the high rates of iron deficiency anemia in infants in Brazil, it is fundamental that the healthcare services should offer adequate prenatal assistance, with the aim of reducing the number of underweight and premature babies, who are more prone to develop various diseases, including anemia.^42^ It is also important that there should be new campaigns to encourage breastfeeding and, after the age of six months, to publicize the need for iron salts to be provided for prophylactic supplementation. Additionally, adequate guidance on diets that emphasize the intake of iron-rich foods such as meat, and also others rich in vitamin C, should be given.^3^

For breastfed babies and young infants between six and 24 months old, iron supplementation is the main form of treatment for iron-deficiency anemia (with a prevalence of at least 20%).^8^ Other actions carry the implication of mid-term and long-term interventions, to be developed by health organizations, such as the fortification of cheap and easily available foods and the diversification of foods promoted by education programs.^7^ Such strategies need to be implemented urgently, especially for the six to 24-month age group.

Supplementation with ferrous sulfate, either intermittently or weekly, is one short-term strategy that can be applied in wide-reaching programs, since it presents two advantages: diminishing the side effects from ferrous salt ingestion and reducing the costs of daily utilization of the mineral.^44^ Food fortification should be utilized as a prophylactic measure, choosing specific foods regularly consumed by the population.^45^ Domestic drinking water has also been used for fortification purposes, giving good results in children.^46^

Dietary guidance is a strategy that should be simultaneously implemented with some other type of program, with the aim of improving the bioavailability of iron. In infants under 24 months of age, higher intake of iron originating from plants is observed, but this iron has lower bioavailability.^47^ Therefore, to improve iron absorption, meat ingestion should be encouraged because it is rich in bioavailable iron, and/or vitamin C should be included when the diet offers low bioavailable iron.

### Programs and strategies developed

Intermittent iron supplementation as a method of controlling anemia is being utilized in Asian countries like Vietnam, Indonesia and China.^16,48,49^ In Brazil, some programs are being developed with the same objective,^5,30,50^ but many more are needed, considering the severity of the problem. Up to 2003, Brazil had been unable to achieve the goal established by the summit meeting in New York,^51^ i.e. to reduce the prevalence of anemia in children and pregnant women by one third.

In 1998, the federal government implemented a prevention and treatment program for iron deficiency anemia in northeastern Brazil, based on a project developed by Coitinho.^52^ This program utilizes the Family Health Program or community health workers, and covers 512 municipalities, with the distribution of ferrous sulfate and guidance on feeding practices for the parents of 300,000 infants between six and 23 months old.^52^ The main objective of this work is to reduce the prevalence of iron deficiency anemia among infants in this age group. Before the program was initiated, 77.5% of the children were anemic. Following the implementation of the treatment program, the prevalence of anemia fell to 40.3%.^53^

The fortification of wheat and corn flour with iron and folic acid is another strategy that was approved by the Brazilian federal government in 2002. Every 100 grams of wheat and corn flour must contain 4.2 milligrams of iron and 150 micrograms of folic acid.^54^

By 2000, the State of São Paulo had already implemented a supplementation project called "Vivaleite", supplying milk fortified with iron to infants, and in particular to under 2-year-olds participating in government social projects.^55^

The fortification of liquid milk with 3 mg of iron has also been utilized in the city of Angatuba, State of São Paulo, for the prevention and treatment of iron deficiency anemia among children under 4 years of age. Iron amino acid chelate was used. In that study 269 children were followed up over a 12-month period. During this period, each child received one liter of fortified milk per day. The prevalence of anemia dropped from 62.3% to 26.4%.^56^

Fortification of domestic drinking water using iron and ascorbic acid has been tested on 21 families of low socioeconomic class in the city of Ribeirão Preto, State of São Paulo. The children and adults drank the water over a four-month period. The increase in hemoglobin concentration in the children was 0.8 g/dl by the end of the study.^46^ This form of fortification might be a simple and effective alternative for treating patients with anemia in less developed regions.

Another form of fortification performed in the cities of Poá and Mogi das Cruzes, State of São Paulo, in 2002, involved preschool children. Rice fortified with iron amino acid chelate was utilized in a proportion of 6 milligrams of iron to 100 grams of rice. The prevalence of anemia dropped from 40.6% to 25% over a 3-month trial period.^57^

In the city of Barueri, São Paulo State, the fortification of bread rolls using iron amino acid chelate was used in the diet of preschool children. Over a two-month period the prevalence was reduced from 37% to 13%.^58^

Cookies fortified with concentrated bovine hemoglobin were introduced to the diet of pre-school children in the city of Teresina, State of Piauí. Over the 3-month trial period, the prevalence of anemia diminished from 75% to zero.^59^

Brunken proposed a program of weekly iron supplementation for the control of anemia in children from 48 to 59 months old. The program was used in the city of São Paulo from September 1995 to September 1996. The amount used was 4 mg/kg per week, over a six-month trial period, for two different study groups. The parents of the first group (control group) were told what their children's hemoglobin levels were and they were encouraged to increase the sources of iron in their children's diet. The parents of the second group (intervention group) received the same information plus a syrup containing iron sulfate to given to their children. The prevalence of anemic children in the intervention group was reduced by 50%.^5^

Another proposal to control anemia was developed in eight municipal crèches in the city of São Paulo from June 1999 to December 2000, among 334 children from six to 36 months of age. Iron amino acid chelate was used as a therapeutic supplement (daily dose of 50 mg of elemental iron) and prophylaxis (weekly dose of 50 mg of elemental iron). The reduction in prevalence of anemia was by 86.5%.^50^

The programs illustrated above show how interventions, even though simple and relatively cheap, can produce highly profitable results, in terms of reducing and controlling iron deficiency anemia among children. All the control strategies for this anemia need to be well targeted and their implementation should be encouraged. The integration of all sectors of society is important in the combating of iron deficiency anemia. Government and private institutions, entities representing society, churches, non-governmental organizations, the general population, health professionals, the media, industries and academic and research institutions are all important for success in controlling this deficiency.

When infants are under 24 months of age, which corresponds to the maturation phase of the central nervous system, they cannot be exposed to the consequences of iron deficiency anemia. The adverse effects from anemia especially affect the development of the neuropsychomotor system, with severe consequences for the future. Thus, combating iron deficiency anemia should be a priority, making every endeavor towards implementing adequate public policies, strengthening community actions, promoting people's involvement and reformulating the healthcare services, with the aim of eradicating this disease.
